# Patient Satisfaction in Medicine and Dentistry

**DOI:** 10.1155/2020/6621848

**Published:** 2020-12-29

**Authors:** Kelvin I. Afrashtehfar, Mansour K. A. Assery, S. Ross Bryant

**Affiliations:** ^1^Division of Restorative Dental Sciences, College of Dentistry, Ajman University, 346 Ajman, Ajman, UAE; ^2^Department of Reconstructive Dentistry and Gerodontology, School of Dental Medicine, Faculty of Medicine, University of Bern, Bern 3010, Switzerland; ^3^Vicerrectorado de Investigación-EIDUCAM, Universidad Católica San Antonio de Murcia (UCAM), Murcia 30107, Spain; ^4^Department of Oral Surgery and Stomatology, School of Dental Medicine, Faculty of Medicine, University of Bern, Bern 3010, Switzerland; ^5^Department of Restorative Dentistry, Edinburgh Dental Institute, College of Medicine and Veterinary Medicine, University of Edinburgh, Edinburgh EH3 9HA, UK; ^6^Centre of Medical and Bio-allied Health Sciences Research (CMBHSR), Ajman University, Dubai, UAE; ^7^Department of Prosthodontics, College of Dentistry, Riyadh Elm University, Riyadh 12611, Saudi Arabia; ^8^Postgraduate Studies and Scientific Research, Riyadh Elm University, Riyadh 12611, Saudi Arabia; ^9^Department of Oral Health Sciences, Faculty of Dentistry, University of British Columbia, Vancouver BC V6T 1Z3, Canada; ^10^Division of Prosthodontics and Geriatric Dentistry, Faculty of Dentistry, University of British Columbia, Vancouver BC V6T 1Z3, Canada

## Abstract

Health professionals, such as medical and dental clinicians, have scant understanding of patients' experiences and perceptions of satisfaction. Nevertheless, implementing a patient-reported outcome measures (PROMs) research practice in surgical sciences is necessary. Hence, the objective of this article was to better understand patients' satisfaction with their medical and dental care. The methods of the current article are based on a narrative review of the literature strategy. A literature review was conducted using both EMBASE and Medline databases up to July 12, 2020, by combining keywords and terms related to “satisfaction theories” and “patient satisfaction,” and “medicine” or “dentistry/stomatology/odontology.” Patient satisfaction's multidimensional nature has been established since the perceived reasons for satisfaction varied widely among patients. Many aspects of treatment influence participant satisfaction at different stages of the intervention process. An improved understanding of the basis for managing patients' expectations with information reiteratively and efficiently may ultimately reduce patients' potential for negative feelings toward the medical and dental treatment experience. Lastly, the consumerist method may misrepresent the still undertheorized concept of satisfaction in health service.

## 1. Introduction

It has been pointed out that early reports on patient-reported outcome measures (PROMs) focused on general patient satisfaction and may not serve to adequately assess the range of impacts of treatment outcomes as perceived by patients [1–5]. Thus, researchers have recommended adding more PROM-related detailed questions to give an insight into a broader range of aspects that might affect patient satisfaction [1]. Nevertheless, there is insufficient understanding of patients' satisfaction by health professionals. Moreover, even the definition of patient satisfaction has not reached consensus. Therefore, the aim of this manuscript focused on three points: to understand the competing aspects of the definition of patient satisfaction; report the main dimensions that influence patient satisfaction; and describe the most relevant theories of satisfaction that have been used in the fields related to surgical sciences, such as medicine and dentistry or odontology.

## 2. Materials and Methods

A narrative review approach was used for fulfilling the objectives of the present study. An electronic search was conducted aided by EMBASE/Ovid and Medline/PubMed engines by combining keywords and Emtree/MeSH terms related to “satisfaction theories” and “patient satisfaction,” and “medicine” or “dentistry/stomatology/odontology.” The search that supported the literature review was carried out up to July 12, 2020. This was complemented by manual searching of the references of relevant studies. Fifty-eight studies met the inclusion criteria; however, not all studies were considered as there was an overlap with some of the secondary sources.

## 3. Results

### 3.1. Definition of Patient Satisfaction

In the last two decades, healthcare provision systems have evolved to be more aligned with patient-centered care [6]. As early as the 1960s, the fields of marketing and healthcare started collaborating on understanding patient satisfaction [7]. Some of the concepts related to patient satisfaction found in the literature are described below:

- The consumer-satisfaction literature describes satisfaction through the lens of a consumer's subjective decision, which relates to his or her expectation and a definite understanding of the merchandise or service [8].
- Patient-satisfaction literature addresses the fulfillment of patients' needs, desires, or expectations in relation to a healthcare service [9]. In this case, the “patient” is an individual user under the guidance of professionals who inform and treat the individual for his/her own sake [8]. As such, “patient” is an appropriate word to be used in studies of healthcare-service satisfaction. However, this concept is somewhat confusing and conflicting in the literature, as it is also increasingly recognized as being multidimensional [7]. The lack of a universally recognized description of patient satisfaction may be related to confusion over validity in quantifying and scoring particular services or experiences [10].
- Patient satisfaction is also frequently used as an important multidimensional indicator in the assessment of healthcare provision quality [7] where patients have quality assurance roles such as contributor (i.e., as a medium of information that permits others its evaluation, to define and evaluate quality), target (i.e., as mediums of control and coproducers of care), and reformer (i.e., by political action, managerial support, through markets, and direct participation) [11].

### 3.2. Importance of Patient Satisfaction

The quality of healthcare provision can be improved by detecting its current problems [12], and a crucial area in which to recognize such problems is the assessment of patient satisfaction [7]. The contemplation of patients' opinions helps to establish appropriate policies and administrative practices, as well as prioritize resource allocation [13]. A high level of patient satisfaction is proposed to arise from prioritizing the patient's views in selecting a health service, attending health professional appointments, and selecting from among suggested therapy options [14]. However, myriad complexities remain in achieving patient satisfaction and the theoretical basis for defining and measuring it.

The following sections will depict satisfaction measurements and satisfaction theories.

### 3.3. Patient-Satisfaction Measurements

#### 3.3.1. Background

Patient-satisfaction measurements are an essential feature in evaluating the quality and effectiveness of healthcare systems, including the assessment of treatment outcomes [15]. In the habitually service-oriented dental medicine approach, patient-satisfaction concepts require a valid theoretical basis for understanding and designing tools for their measurement [16].

Two decades ago, a review of dental patient satisfaction evidenced that the common dimensions (also known as “domains” or “determinants”) contained in patient-satisfaction surveys were concerned with operator perceived skills, interpersonal aspects, convenience, finances, and the clinical environment [16]. Once the multidimensionality of patient satisfaction was acknowledged, multi-item surveys were developed with a view to measuring or evaluating satisfaction in a convincing way. As the measurement of patient satisfaction evolved, global healthcare instruments (e.g., the Medical Interview Satisfaction Survey [17] and the Medical Satisfaction Questionnaire [15], among others) served to inform the critical domains considered in oral health tools [18, 19]. These medical patient-satisfaction questionnaires were adapted to other health sciences areas. For this reason, oral-health-patient-satisfaction questionnaires were developed with inconsistent conceptual dimensions [16]. A recent review of patient-satisfaction questionnaires in healthcare has documented the evolution of various dimensions utilized through decades of development (Table 1) [20].

#### 3.3.2. Patients' Subjective Perspective in Satisfaction Tools

Considering the patient's subjective perception is imperative for the soundness of the instruments evaluating satisfaction since patient satisfaction is a crucial feature of the assessment of healthcare services [15]. A recent critical review [20] assessed 14 dental patient-satisfaction instruments that underwent psychometric validation with 8 to 42 items and 2 to 13 dimensions. However, the review reported that methodologies to integrate a patient's subjective perspective were missing [20].

The patient-satisfaction tools used in subsequent follow-up studies that tested for validity were the Dental Visit Satisfaction Survey (DVSS) [21, 22] and the Dental Satisfaction Questionnaire (DSQ) [23–25]. The DVSS [18] evaluates satisfaction regarding a particular dental visit, whereas the DSQ [19] evaluates a healthcare system's global perspective. The internal consistency (alpha) ranged from 0.86 to 0.89 and 0.77 to 0.81 for the DVSS [21, 22] and DSQ tools [23–25], respectively. Although this implies that the tools contain items that could be equivalent measurements with satisfactory unidimensionality for scale composition [26], these studies barely followed the guidelines of the COSMIN checklist (Consensus-based Standards for the Selection of Health Status Measurement Instruments) [27], which appraise the methodological quality of studies on the measurement properties of PROMs. Consequently, the level of validity and reliability of the DVSS and DSQ tools must, unfortunately, still be considered uncertain.

#### 3.3.3. Potential Solutions for Current Weaknesses of Satisfaction Measurements

A recent systematic review of the determinants of patient satisfaction [28] recommended producing new studies with standardized surveys that are adjustable to particular populations for supplementary comparisons. The authors recommended that these studies could consider cultural, behavioural, and socioeconomic disparities to determine their impact on patient satisfaction. To reduce bias, valid instruments could have open questions for patients' commentaries and criticisms. Moreover, to detect true causal relationships, the type of research design would optimally be longitudinal or experimental [28].

#### 3.3.4. Importance of Determinants Evaluated in Satisfaction Instruments

There is a need for evidence of the effects of potential health-related and patient-related determinants in shaping patient satisfaction [29]. Healthcare-service quality indicators are regarded as the most prominent determinants of patient satisfaction [28]. Among these, interpersonal care is considered a crucial determinant. However, patient sociodemographic factors are contemplated only as potential determinants and confounders since their associations with patient satisfaction have been inconsistent [16]. Thus, a standardized patient-satisfaction questionnaire to obtain information on cultural, behavioural, and sociodemographic disparities has been proposed to provide more consistent associations [28].

In conclusion, the dimensions of patient satisfaction important to patients can be documented by studying patient experience and perceptions. Such a study can provide a valid theoretical basis for developing or initially evaluating a patient-satisfaction questionnaire that may be more valid for the quantitative scientific study of the phenomenon.

#### 3.3.5. Limitations of Satisfaction Measurements

When expectations are applied as a satisfaction gauge, it should be indispensable to determine which type and level of expectation (e.g., expectancy probability, predicted, unformed or partly formed, normative, process and outcome expectations, among others) are held by the patient [30]. By having more than one concept of patient satisfaction, the comparability among studies is reduced since they used varied measurement instruments, which contemplated dissimilar dimensions [7, 28, 31–33].

Moreover, the contradictory evidence about potential determinants among patient-satisfaction studies severely impacts the internal and external validity of the findings [28]. The most influential factors that impact patient satisfaction remain inconclusive [34]. Thus, the limitations may be due to a lack of consensus on the theoretical framework of patient satisfaction [33, 35] and the multifaceted concept of patient satisfaction with several causal aspects [7, 18, 19, 28, 31, 32, 36].

### 3.4. Customer-Satisfaction Theories

The most widespread application of the concept of satisfaction has been related to the understanding of customer satisfaction in the sale of products or services. The notion of satisfaction is also prevalent in marketing theory, which is rooted in business models and, ultimately, in the concept of consumerism. Moreover, service quality's discernment has been shown to arise from the potential discord between customer expectations and customer experiences [37, 38].

**Figure 1:**
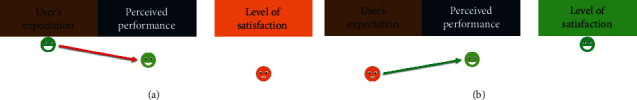
(a) Negative disconfirmation or dissatisfaction and (b) positive disconfirmation or satisfaction in the user's satisfaction theory.

The theoretical basis of such consumer satisfaction is the user's satisfaction theory [39, 40]. The user's satisfaction theory is an expectations-based theory that states that in spite of a person having had a positive experience with a service or product, they may still end up dissatisfied if the experience did not meet or exceed their original expectations (producing what is termed negative disconfirmation) (Figure 1(a)). On the other hand, satisfaction would result from the experience of a product or service outperforming the expectations (producing what is termed positive disconfirmation) (Figure 1(b)) [41, 42].

Thus, satisfaction is conceptualized as an emotion arising from the user's assessment of the perceived performance relative to his or her expectation, where it might otherwise have been mistakenly seen as only an emotion [43]. In addition, user anticipation about the level of performance that will be delivered by a product gives foundation to his/her expectations [41]. In other words, it is not essential in the model for expectations to start with a negative to achieve satisfaction, nor for perceived performance to end negative to yield dissatisfaction; only the relative differences between expectations and performance impact the outcome in this model. This is also known as the Disconfirmation Model [41].

In contrast, assimilation theory recognizes that consumers seek to avoid dissatisfaction by modifying their perceptions about a product or service to make it more comparable with their expectations [44, 45]. This would diminish consumers' distress from what would otherwise be negative disconfirmation, and consumers may have more than one mechanism to accomplish this. They may lower their expectations enough to match their experienced product performance. Or, the dissatisfaction that consumers experience from a disagreement between their expectations and perceived performance of a product could be reduced by belittling the meaning of the initial dissatisfaction.

The tolerance level concept proposes that customers are prepared to accede to a range of performance outcomes from the service or product, provided the range can be realistically estimated [46]. The next section will provide specific theories that have been adapted to concepts of patient satisfaction in professional health settings.

### 3.5. Patient-Satisfaction Theories

Most patient-satisfaction theories appear to have been borrowed from the fields of consumerism or marketing and inserted into the healthcare literature with minimum adaptation [7]. A recent metanarrative review by Batbaatar et al. [7] reported on what is currently known about the conceptualization of patient satisfaction. The review established that, unlike in marketing and consumer theories, there is only a vague, or perhaps at least an inconsistent, relationship between expectations and patient satisfaction. The noncritical utilization of marketing theories obscures their full transferability to the health field since customer and patient satisfaction are likely dissimilar concepts. The authors concluded that the patient-satisfaction concept needs to be better defined and distinguished from other perspectives, preferably consistent with how patients evaluate their experiences rather than using consumerist theories. There is evidently still a need to improve our understanding of how patients assess the care they receive [7]. Thus, patient satisfaction is an undertheorized concept [47]. In addition, the concept of expectations has not clearly been theorized in relation to patient satisfaction [7].

To date, patient satisfaction conceptualizations largely rely on the interactions between expectations and perceptions as described in consumer or user's satisfaction theory [8]. Interestingly, marketing research has also incorporated psychology concepts, as expectation theories were first established in that field [48]. Leading from this one school of thought to conceptualize patient satisfaction, expectations are considered the most important aspect of patient satisfaction. This is based on the principle in expectation theories that describes patient satisfaction purely as a consequence of how satisfactorily a health service met patient expectations [49]. Overall, the theoretical associations concerning patient expectations and satisfaction remain uncertain [50]. Healthcare has also implemented some theories from service literature in an attempt to rationalize patient satisfaction through an association with expectations [48]. When patient satisfaction is analyzed, the effects of “assimilation and contrast” and “zone of tolerance” on patients' subjective values should be considered [32, 51–53]. The most significant theories on patient satisfaction originating in the field of consumer-satisfaction theory [7] follow in alphabetical order:

#### 3.5.1. Attribution Theory

Based on the user's satisfaction theory, attribution theory attempts to clarify the root of discordancy between expectations and experiences. In this instance, dissatisfaction results from unmet expectations (Figure 1(a)) [16, 51, 54]. Nevertheless, patients and healthcare providers can have dissimilar explanations for not satisfying patient expectations. Thus, this theory primarily deciphers patients' understanding of events in addition to the origins of understanding their behaviour [51].

#### 3.5.2. Discrepancy Theory

This theory, which along with discrepancy theory is also based on the user's satisfaction theory, posits that both the level and route of dissimilarity between a therapy outcome and the expectations around it determines a consumer's satisfaction [51]. For instance, if satisfactory outcomes endorse positive expectations or disconfirm negative expectations, it results in satisfaction [30]. It also explains the difficulty in perceived outcomes surpassing expectations that are sustained at a high level, thus being less likely to yield high satisfaction [55]. Interestingly, based on expectations, the best theory to illuminate the relationship between expectations and satisfaction has been the disconfirmation paradigm [56].

This theory's primary limitation is the same as any other expectations-based theory: it lacks adequate consideration of a multidimensional concept of satisfaction.

#### 3.5.3. Disconfirmation Theory

This theory holds expectations as the baseline, and satisfaction is evaluated as proportionate to the difference between patients' expectations and experience [48]. Thus, satisfaction displays an inverse association with any discrepancy from expectation, regardless of it being positive or negative [57, 58].

This theory has been criticized for describing dissatisfaction as simply the divergence between expectations and experiences, which does not take into account that a seemingly favourable divergence could generate a counterintuitive result [48].

#### 3.5.4. Economic Theory

The economic theory states that patients expect to receive healthcare services of equivalent or better quality relative to the fee charged for the delivered service [55].

#### 3.5.5. Equity Theories

These theories affirm that a patient seeks to match the value of the outcome obtained by other individuals; thus, they are associated with social comparison theory. If the patient believes that both input and output ratios are reasonable for the healthcare service, then satisfaction is achieved. The “input” refers to resources (e.g., money, time, pain) and the “output” to the outcome itself (i.e., health improvement) [50].

#### 3.5.6. Healthcare Quality Theory

This theory proposes that a satisfactory opinion of several features focused on patient views of quality care is paramount in determining patient satisfaction. Interestingly, interpersonal care is considered an extremely influential factor in satisfaction [53].

#### 3.5.7. Holistic Approaches

**Figure 2:**
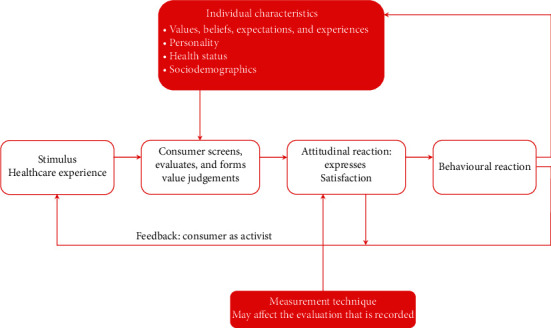
Holistic model of satisfaction with healthcare (adapted from Strasser and Davis [59] and Strasser et al. [60]).

The concept of global, or holistic, satisfaction represents a combined feeling resulting from positive or negative emotional reactions to what could be several domains of healthcare service that influence patients' assessments (Figure 2) [59, 60].

In spite of the fairly widespread use of such global indicators to capture patient satisfaction, studies have unfailingly recognized that patient satisfaction is multidimensional in nature and that the dimensions taken into account in assessing patient satisfaction fluctuate from study to study [55].

#### 3.5.8. Multiple Models Theory

Three autonomous models of patient satisfaction have been formulated where numerous factors shape the concepts of satisfaction, thus recognizing satisfaction is not a single concept [61].

- One model explained that psychosocial differences shape both expectations and satisfaction
- The next model outlines that the ultimate assessment for some patients is not actually satisfaction but the accomplishment of health objectives aided by healthcare services
- The third model suggests that certain health problems cause emotional uneasiness that eventually prevents patients from reaching satisfaction

The proponents concluded overall that patients' expectations are highly influenced by both [61] patients' assumptions of potential health outcomes and the level of disruption of their self-sense by their affliction and healthcare delivery.

As a limitation, these models have been criticized as being vague [32].

#### 3.5.9. Need Theory

Need theory proposes that prioritization of healthcare objectives from the standpoint of both the clinician and the patient allows visualization and understanding of their discrepancies (Table 2) [62]. For example, whereas patients may initially choose feeling well, clinicians tend to habitually follow a strategy based on four levels of assessment and management objectives. While the theory proposes simplicity in the framework's order, variations in the proposed order are anticipated.

**Figure 3:**
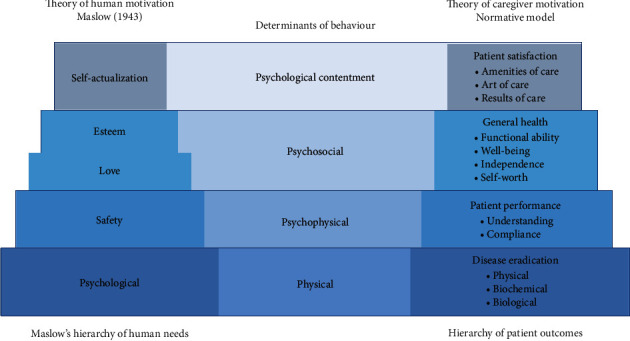
Human needs and patient outcomes construct (adapted from Maslow [63] and Johnson [64]).

Need theory supposes that patients' needs are equivalent to patients' expectations [53]. The root of this understanding is Maslow's human motivation theory [63]. Thus, a patient's process of achievement of each level of Maslow's hierarchy of needs would influence the level of patient satisfaction directly. Once self-actualization is achieved (Figure 3), the patient is considered to be pleased with the healthcare service. When all of a patient's psychophysical needs are met, then the concluding need of the hierarchy is achieved.

Clinicians are thought to facilitate this process by understanding their patients' needs, each with their own characteristics, pathologies, and healthcare experiences. In other words, needs differ substantially from patient to patient [64].

To further understand how patient satisfaction is developed, researchers contrasted two theories, Maslow's hierarchy of needs [63] and the hierarchy of patient needs, which was based on a normative model using the theory of caregiver motivation [64]. The hierarchy of patient needs catalogues patient outcomes in four categories, building from a base focused on physical needs, as shown in Figure 3 [64]. With that understanding, thereafter, some items of Maslow's hierarchy of needs were seen to be parallel to the hierarchy of patient needs. Moreover, self-actualization was considered the most significant determinant of enthusiasm and is only achieved once all other human needs are met (Figure 3). By the same token, patient satisfaction is a critical goal for clinicians and is only achieved once all patient outcomes are fulfilled [64].

#### 3.5.10. Value Expectancy Model

The need to understand people's views of their motivations and actions gave birth to the expectancy-value theory. This theory further evolved into the value expectancy model through the inclusion of the assessment of five psychosocial variables (e.g., expectations) that may impact patient satisfaction [52].

One of the main issues with this model is how it conceptualizes patient satisfaction, given that satisfaction seems to be more influenced by a rapid response to the healthcare experience, rather than a patient's previous expectations and common values [48].

### 3.6. Important Considerations for Understanding Patient Satisfaction

It can be understood that human expectations are predisposed by personal characteristics, environmental aspects, and previous experiences [7–10]. A successful service is more likely to be provided when patients' expectations are recognized at the commencement of treatment [10]. Conversely, patient expectations may modify somewhat during the course of treatment [65] since it has also been recognized that expectations are situation-specific.

**Figure 4:**
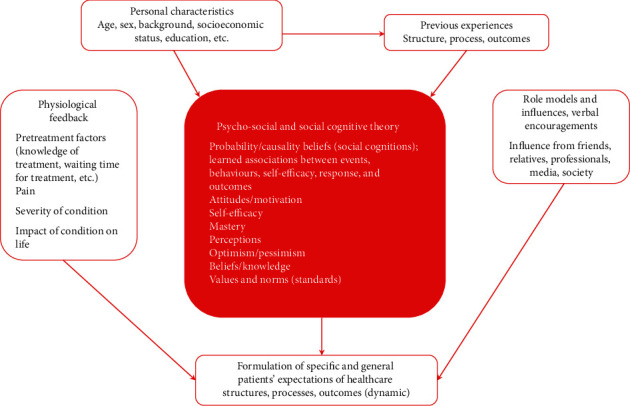
Model based on the literature of multiple influences on patients' healthcare expectations (adapted from Bowling 2012 [26]).

Most of the patient-satisfaction theories and models described here, except for the health quality and holistic theories, rely on specific concepts of patient expectations. In spite of extensive theoretical development, measures using expectations are not good predictors of patient satisfaction with treatment outcomes. The limitations of the expectations-based approaches have been well recognized, spurring further efforts to develop patient-satisfaction concepts, including a health-service components approach [55]. This approach elucidated the multidimensional notion of satisfaction being guided by several internal and external features of healthcare-service delivery [66]. The concept of “expectation” is dynamic and multidimensional, influenced by patients' characteristics, including their belief system, preceding experiences, and pretreatment situation [53]. Subsequently, it has been recognized that using expectations to elucidate satisfaction is problematic (Figure 4) [30].

## 4. Discussion

The review of patient-satisfaction literature here found a poorly related relationship amongst patient expectations and satisfaction, as well as that expectations relate only poorly to variations in satisfaction [30].

Expectations may not explain satisfaction for a number of reasons:

- The connection between expectations and satisfaction is not well understood, and they may not be linked in an explicable way [50]. Satisfaction may be only indirectly influenced by expectations instead of directly [67]. Previous information and experiences, as well as patients' characteristics (e.g., socioeconomic status, values, other conditions), may influence the range of diverse kinds of expectations in society [32].
- The way expectations are commonly communicated is implied and speculative [53]. In addition, it is challenging to measure both expectations and experiences in a valid manner [55].
- Researchers cannot rationalize patients' expectations about health services and how comfortable they feel sharing this information if they decide to share it [7]. Hence, researchers are still far from understanding how patients cultivate expectations and the manner in which they express them [53].
- The consumerist method may misrepresent the concept of satisfaction in health service [7]. Therefore, the undertheorized patient satisfaction concept needs to be better defined and consistent with how patients evaluate their experiences.

## 5. Conclusion

Existing patient-satisfaction questionnaires have been noncritically borrowed from marketing theories, so it is not surprising that they have not been that useful in health fields, since customer and patient satisfaction are likely dissimilar concepts. Therefore, the patient-satisfaction concept needs to be better defined from other perspectives, preferably consistent with how patients evaluate their experiences rather than by presuming to rely on consumerist or marketing theories. In itself, the notion of patient satisfaction being multidimensional can also be considered a theory or at least a theoretical or conceptual basis.

## Figures and Tables

**Table 1:** Dimensions of tools used for appraising patient-satisfaction in healthcare in descending order of time appearance (adapted from Nair et al. [20]).

Year	Dimensions, *(N)*	Dimensions of patient satisfaction
2009	4	Clinical atmosphere, treatment process, care outcome, and cost
2007	4	Treatment, communication, clinic, and appearance
2007	2	Belief about care and atmosphere
1997	3	Access, communication, and quality
1996	10+	Communication, services received, care outcome, staff, waiting time, clinic location, appointments, dental professional, affordability, and conceptually unrelated items
1995	8+	Communication, services received, care outcome, staff, waiting time, clinic location, appointments, and conceptually unrelated items
1985	13	Dentist-patient relations, technical quality of care, access, waiting time, cost, clinic, availability, continuity, pain, staff perform expanded duties, staff-patient relations, staff technical quality, and clinical atmosphere
1984	3	Communication, understanding-acceptance, technical competence
1981	6+	Access, availability, pain, cost, quality, and conceptually unrelated items
1978	2	Latent hostility and general glorification
1975	3	Cost, convenience, and quality
1974	3	Personality, technical ability, clinic, and cost

**Table 2:** Healthcare objectives from clinicians and patients' perspectives (adapted from Kvale [62]).

Healthcare objectives		
Priority	Clinicians' perspective	Patients' objectives
1	Determining the etiology of the disease	Feeling well
2	Understanding the presenting symptoms and clinical signs	Being able to function
3	Improving the patients' ability to function	Improving clinical signs and symptoms
4	Improving the patients' sense of well-being	Understanding the etiology of the disease
